# The dynamics of intergenerational closure and family networks of social cohesion

**DOI:** 10.3389/fsoc.2023.933216

**Published:** 2023-03-02

**Authors:** Michael Windzio, Patrick Kaminski

**Affiliations:** ^1^SOCIUM, University of Bremen, Bremen, Germany; ^2^Department of Sociology, Indiana University, Bloomington, IN, United States; ^3^Luddy School of Informatics, Computing, and Engineering, Indiana University, Bloomington, IN, United States

**Keywords:** intergenerational closure, school networks, stochastic actor oriented model, social cohesion, families

## Abstract

We investigate the correlation of ties among school-children's parents with violence in schools, and two mechanisms of intergenerational closure (IC). Coleman described ties among parents of befriended children as IC. Until now, IC indicated social capital in schools and neighborhoods, but existing evidence is rather ambiguous and does not utilize network data. According to “top-down.” IC, children establish network ties because of the acquaintance among their parents. “Bottom-up” IC implies that children make friends first and then their parents get involved. We use longitudinal social network data from *k* = 10 school classes and *N* = 238 adolescents and disentangle the two different dynamics of IC by applying Bayesian stochastic actor-oriented models (SAOMs) for network evolution. SAOMs show positive “top-down” and “bottom-up” effects on IC, with the latter being considerably stronger.

## 1. Introduction

In our study we elaborate two different mechanisms of social network closure across generations and argue that intergenerational closure (IC) relates to violent behavior in schools. The concept of IC (Coleman, 1987) describes network ties among families of befriended children, which contribute to social cohesion at local levels, such as schools or neighborhoods. Studying social cohesion and solidarity has a long tradition in sociology. While Max Weber elaborated his view on social order and legitimacy (Weber, 1978), Emile Durkheim (1965) analyzed the change from mechanic to organic solidarity, as well as the effect of elementary forms of religion on social integration (Durkheim, 1915). Social cohesion resurged as an important issue in post-industrial and culturally diverse societies (Schaeffer, 2014), particularly in periods of increasing economic inequality (Hung, 2021). Yet, the conceptual elaboration and empirical measurement of social cohesion continues to pose a challenge. Social cohesion is not a characteristic of individuals, but of relations among them in a social system, e.g., in families. Cohesion is a *relational* concept and empirical research on relational concepts requires relational data, such as data on social networks, or data on the relationship between individuals and social systems, e.g., organizations, families or other kinds of groups (Lawler et al., 2008). The theoretical conceptualization and the empirical measurement of cohesion should elucidate the level of analysis. The lowest level to analyse cohesion is the dyad, the relationship between two persons. Social order exists in dyads, particularly in families, when both actors meet mutual expectations, which ideally results in cooperation and in the creation of collective goods by reciprocal exchange (Trivers, 1985). In addition, dyads, families and small groups are often embedded in higher order social systems, such as neighborhoods, nation states or even supranational organizations. Clarity on the respective levels at which researchers analyse social relationships and cohesion is therefore essential. In many cases, social cohesion results from multilevel processes, when specific forms of relationships in a lower-level social system determine the overall cohesion and cooperation in this system (Bowles and Gintis, 2011), for instance with respect to the generation of collective goods.

James Coleman's concept of “intergenerational closure” in schools and neighborhoods is based on such a multilevel perspective. Intergenerational closure focuses on relations among families and describes a four-cycle network structure in which not only children, but also their parents are linked to each other. Network ties among parents' facilitate the creation, coordination and enforcement of social norms, as well as the handling of children's conduct problems, such as disputes, bullying or aggressive behavior. Research has shown that intergenerational closure (IC) is an important aspect of social capital at the community level, e.g., in schools or in neighborhoods.

Although intergenerational closure refers to a specific network structure among families, many empirical studies based on this concept do not analyse complete networks. Differences between open and closed networks are taken as given, without distinguishing between particular causal mechanisms of closure. Coleman refers to local organizations, e.g., churches or clubs, where parents come together in their neighborhood and get involved within networks. Researchers often focus on a unidirectional mechanism of how intergenerational closure emerges: network ties among parents emerge from membership in local organizations, regarded as exogenously given, independently from children's activities.

In contrast to this unidirectional view, we argue that intergenerational closure depends on two mechanisms: first, children establish network ties due to the acquaintance among their parents. Ties among parents create opportunities to meet, to get to know each other and to become friends. Here, parental networks exist first, and children establish friendship ties afterwards. We call this mechanism “top-down” closure. Second, children make friends first and then their parents get involved due to children's friendships, so children's friendships come first and parental network formation second, which we call “bottom-up” closure. Parents get in contact with parents of their children's friends, e.g., by arranging meetings or picking children up from their friends' homes.

In the first empirical part of our study, we analyse the correlation of the average degree in the parents' networks with the density in children's network of violent behavior. Our results suggest a negative correlation of ties among parents and violent behavior in school. However, the preventive effect of parental ties on violence seems to unfold over time: we found a negative association of IC and violence in the third year of secondary school, whereas this preventive effect of IC was absent before.

In the second step, we use longitudinal social network data from *k* = 10 school classes and *N* = 248 children to disentangle the two dynamics of intergenerational closure, namely “top-down” and “bottom-up.” Results of Bayesian stochastic actor-oriented models (SAOMs) for social network evolution support both mechanisms. We will discuss our results with respect to the theoretical background, measurement error and data quality and give an outlook for further research on intergenerational closure, which we consider crucial for social integration at the level of schools and local communities.

## 2. Intergenerational closure and social capital: Theory and research

Studies on social cohesion and social integration (Putnam, 1992, p. 163) often focus on how local communities create social capital and intensify social integration, e.g., by developing trust relationships in social exchange networks (Blau, 1986; Lawler et al., 2008; Windzio, 2018). What these studies have in common, at least, is that they apply cohesion as *relational, not individual* concepts. The smallest unit for which we can analyse cohesion or integration is the dyad e.g., in cooperation games (Diekmann and Lindenberg, 2001).

**Figure 1:**
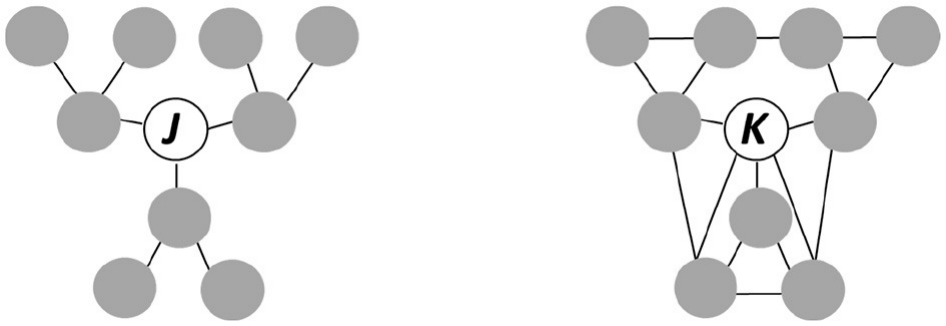
The difference between individual and collective social capital.

Theoretical arguments relate dyadic or triadic micro-level processes to different levels of social cohesion at the macro-level (Lawler et al., 2015). Network processes at the micro-level are crucial elements to build theories of social integration because the cohesion of social systems, e.g., families, school-classes, or neighborhoods, often results from micro-level network-processes. Actors' behavior, in turn, is embedded in network structures. We find the idea of socially embedded behavior already in the classic work of Thomas Hobbes, who suggested “third-party enforcement” as a solution of the cooperation-dilemma (Fiske, 1992, p. 701; Putnam, 1992, p. 165). For instance, if two actors interact with a third actor in a triadic trust game, social control and norm enforcement are only possible if the two trustors communicate about the trustee's behavior (Buskens, 2003). Social capital thus results from specific forms of social organization (Putnam, 2007; Portes and Vickstrom, 2011; van der Meer and Tolsma, 2014)—often from appropriate *institutions*—which facilitate social relationships and thereby contribute to the emergence of norms and trust. Moreover, socially embedded interaction occurs in networks (Glanville and Bienenstock, 2009, p. 1511). A famous example is the diamond market of New York City, where merchandise is worth hundreds of thousands of dollars, and merchants often hand over bags of stones to other merchants who take them home for examination (Coleman, 1988). Here, norm enforcement and trust reside in repeated interaction and informal social control within the ethnic-religious community (Richman, 2006). In contrast to Bourdieu, who regards social capital as an *individual* characteristic (Bourdieu, 1986; Glanville and Bienenstock, 2009; Forrest and Kearns, 2016, p. 2138), Coleman underlines the *collective* benefits of norms, sanctions and trust (Halpern, 2006, p. 17), which are to the benefit of “… all those who are part of such a structure” (Coleman, 1988, p. 116). Figure 1 highlights the multilevel character of network integration by distinguishing between individual and collective social capital.

Actor *J* in Figure 1 is in a broker position. *J* has direct access to alters' resources and can control the flow of information through the network. Moreover, *J* can strategically display different attitudes and loyalty to their respective contacts (Burt, 2001). *J*'s *individual* social capital is comparatively high due to the position in the network. Actor *K*, in contrast, does not have such a strategic position. Compared to other actors, *K*'s level of individual social capital is not considerably higher. Due to network closure (Coleman, 1988, p. 106), however, the degree of embeddedness of each relationship is high, so that *K*'s community can easily generate norms and coordinate norm enforcement by appropriate sanctioning (Coleman, 1988; Burt, 2001). *K* is thus a member of a *community* with high social capital. Structural conditions at the community level, in this case the network topology, determine the opportunities to exchange rights to control the actions of other actors at the dyadic level (Coleman, 1990, p. 242). Dyads, in turn, are embedded in higher order substructures such as triads or four-cycles.

The concept of *intergenerational closure* is a famous example of community-level social capital (Marsden, 2005, p. 9, for an overview). It describes different network structures among families. Coleman argues that “(…) in the course of gossip and casual conversation (…),” parents, and particularly mothers, “(….) would have occasion to discuss contingencies in their children's behavior and activities before they occur. These discussions lead to establishing norms for their children's activities, norms that they know will be reinforced by parents of their children's friends. Maintenance of the norms is in the interest of all parents, and in a sufficiently close functional community, parents will sanction not only their own children, but their children's friends (i.e., their friends' children) as well. (…) Power, which in the absence of intergenerational closure is in the hands of the children (who maintain a thriving functional community in their own age group, facilitated by the school), is in the hands of parents when such closure exists. They are armed with a set of norms and aid one another in the enforcement of the norms” (Coleman, 1987, p. 189).

Once established in this way, the validity of norms is a characteristic of the social system, not of individuals. Individuals can violate a norm, but should expect sanctions from the community. Network structures—open or closed—thus determine the scope of individual action and the collective capacity of creating norms at the community level. Empirical studies, however, often ignore the different mechanisms of intergenerational closure. Scholars underscore the role of local organizations (Glanville, 2004; Fletcher et al., 2006b), e.g., Catholic churches as an ideal case in Coleman's work (Coleman, 1987, 1988), where parents establish their own networks, without considering ties among their children. In these neighborhoods, networks become closed when also their children become friends. In the prototype Catholic school, children befriend their peers whose parents are already acquainted with their own parents. Here, social capital is a result of parents' local associations, which come first, and friendships at the children's level come afterwards. Differences in intergenerational closure between public and Catholic private schools thus indicate different “ideal types” of local social organization. But ties among parents that precede ties among children are only one mechanism of intergenerational closure.

### 2.1. Two mechanisms of intergenerational closure

While studies often take contact among parents as given due to clubs, religious and social organizations in neighborhoods, longitudinal social network analysis is able to disentangle different mechanisms. In Coleman's concept, ties among parents come first and friendship at the children's level afterwards. In public schools, however, children naturally establish friendships with their peers, and often start to meet their friends after school (Windzio, 2015), which is a reason why children's parents become involved, establish contact and *thereby* networks become closed. When it comes to mutual visits at home, parents even take the role of third parties who either facilitate or impede the intensification of their children's friendships (Windzio, 2018).

**Figure 2:**
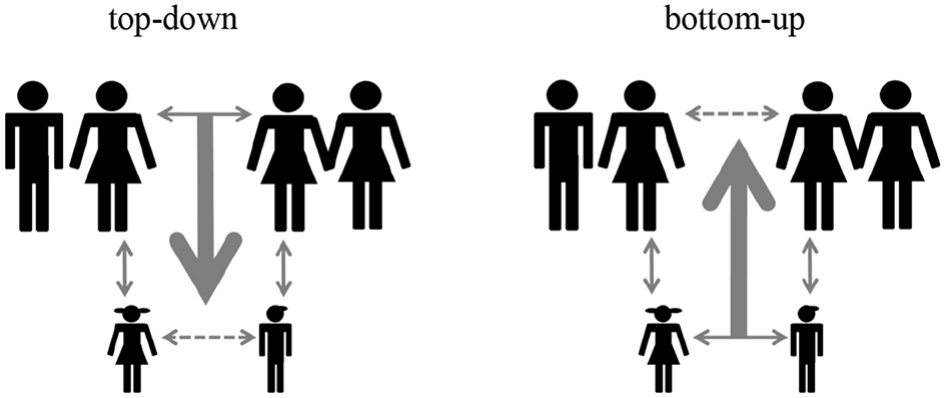
Intergenerational closure results from two different mechanisms.

Figure 2 highlights the two mechanisms of intergenerational closure. Dashed lines indicate the respective outcome of each intergenerational closure mechanism. In the left panel, the “*top-down*” mechanism represents the usual understanding of intergenerational closure in most empirical research as well as in Coleman's original concept. Here, the driving force of closure is the social organization of the community, e.g., by clubs, churches and other focus points where parents are active and get involved. When becoming friends, their parents are already acquainted since they are members in community organizations. According to the top-down mechanism, children's friendships are embedded in ties among their parents right from the beginning. One example of a decline in top-down-closure is the family's residential relocation. Residential mobility dissolves social relationships, particularly among parents (Sampson et al., 1999, p. 645), so that mobile parents do not benefit from local social capital, even if it were available in the community (Coleman, 1988, p. 113).

In contrast, the “*bottom-up*” process might be more prevalent in most public schools in Germany, where pre-organized local communities, such as religious groups and religious private schools (Jungbauer-Gans et al., 2012), are less important than in the U.S. Children become friends and corroborate their relationship during leisure time activities, which often involve their parents. There might be some selective strategies adopted by parents when they try to influence children's friendships, but according to the bottom-up process, parents establish network ties *as a result* of their children's friendships.

### 2.2. Behavioral consequences of intergenerational closure

Analyzing norm violations in the Wikipedia editing process, Piskorski and Gorbatai (2011) found positive effects of network closure on norm compliance, which is in line with Coleman's (1990) general argument. However, empirical evidence regarding the behavioral *consequences of IC* is ambiguous (Halpern, 2006, p. 152). Differences in IC cannot explain differences in mean levels of mathematics achievement between public and Catholic private schools. Conditional on the density of children's friendship networks, closure even has a negative effect on gains in mathematics achievement (Morgan and Sørensen, 1999). Accordingly, the benefit of closure does not necessarily out-weight its costs, which are e.g., tightly knit but isolated communities, parochialism, “negative” social capital (Portes, 1998), as well as little novel and diverse information, which would be otherwise accessible through weak tie networks (Granovetter, 1973; Coleman, 1987, p. 190). Other studies, in contrast, find positive effects of closure on educational outcomes (Carbonaro, 1998; Kao and Rutherford, 2007). Under some favorable conditions, IC in schools and neighborhoods has positive effects on children's wellbeing and academic performance (Fletcher et al., 2006a). In a recent study using panel data, Geven and van de Werfhorst (2020) did not find effects of IC within individuals on grades. In other words, a change in the exposure to closure does not significantly increase grades *within* the same individual. The only effect is *between* individuals, which the authors attribute to unobserved time-constant heterogeneity between students (e.g., unmeasured traits), which can bias between-, but not the (insignificant) within-estimates in their panel model (Geven and van de Werfhorst, 2020, p. 48).

IC can be distinguished into weak and strong ties, e.g., when survey items ask whether persons either “Have talked to each other a few times” or “Chat or meet a lot” (Chang, 2009). According to Chang (2009) strong parental closure around schools has no consistent effect, whereas weak parental closure has consistently positive effects on cognitive ability in higher secondary schools. These effects are less systematic and mostly insignificant in junior high schools (Chang, 2009). Intergenerational closure is also ambivalent with respect to teenage sexual behavior, since it reduces inconsistent condom use (Moore, 2010, p. 35), but does not have an effect on the number of sexual partners (Moore, 2010, p. 33).

IC is an issue also in criminology, where it describes a mechanism of how communities create social capital and enhance informal social control (Sampson et al., 1999; Oberwittler, 2004; Windzio and Heiberger, 2022). Following Coleman's argument, IC might affect delinquency and violence in neighborhoods or schools. Regarding Afro-American boys, Mangino (2009) showed that adolescents who are in the position of social bridges between groups tend to be less delinquent than those who exclusively belong only to one peer group. This result is explained by the stronger influence of parents in more *dis*connected peer networks (Mangino, 2009). For the Netherlands, Dijkstra et al. (2004) tested the functional effect of communities around high-schools, and found a negative effect of IC on delinquency, even though the effect was rather small. Another study showed that collective efficacy significantly reduces delinquency, whereas the effect of intergenerational closure is even *positive* and marginally significant when controlling for collective efficacy (Valasik and Barton, 2017). This result challenges an important proposition derived from the IC concept: “… if neighborhood residents use the networks and associations between neighbors for the benefit of the common good then there should be a reduction in the amount of destabilizing behaviors that take place” (Valasik and Barton, 2017, p. 1658). The authors conclude from their findings that while collective efficacy is a rather global measure of social capital, intergenerational closure might work at a rather local level. In a recent study, Windzio and Heiberger (2022) analyze the embeddedness of school-children's ties in parents' networks, and also show that the school's environment moderates the preventive effect of IC on violent behavior.

The present study analyses social networks in school contexts. Our empirical model is focused on local-level school-class networks and we expect that school classes where intergenerational closure is high are more resilient to school violence. Our approach to analyzing the effects of IC is to directly estimate the prevalence of closure in a set of social networks. Longitudinal social network data provides a direct measurement of IC, as suggested in Coleman's work, and also the causal mechanisms generating closure can be identified.

Previous research has shown that the degree of ethnic boundary blurring (Alba and Nee, 2003) in networks considerably differs between children and parents. While children easily make interethnic friendships in schools, parents of immigrant children do not have similar opportunities to befriend adults from the outgroup. As a result, social networks of parents are much stronger segregated along ethnic lines than children's networks (Windzio, 2012; 2015). We thus control for (non-)immigrant status in our analysis of “bottom-up” or “top-down” intergenerational closure.

## 3. Data and methods

We use 3-wave-network data collected in public schools (Windzio, 2018) in the years 2010, 2011, and 2012. Response rates of students varied from 75.4% in wave 1 to 80.4% in wave 3. Since the participation of schools depended on the school principals' consent, and also on the teachers' willingness to support this study, there was considerable non-response at the school- and class-level, so that one third of all classes in the population did not participate. Since the quality of social network data depends on participation rates within classes, only classes where either 75% or *N* = 17 students participated have been analyzed. The descriptive analysis (**Figure 4**) was limited to classes participating in all three waves, so that *N* = 501 in 21 school classes were available for the analysis. In contrast, in the SAOM analysis only 10 classes and *N* = 248 adolescents could be used due to problems of model convergence, which is a consequence of the low density of the parental contact network (see Table 1).

**Table 1:** Descriptive statistics, of *k* = 10 classes, *N* = 248 students.

	**Wording**	**Time**	**Mean**	**SD**	**Min**	**Max**
**Network densities**						
Friendship	Which classmates are your friends?	1	0.213	0.062	–	–
Friendship		2	0.241	0.094	–	–
Friendship		3	0.234	0.089	–	–
Parental contact	Do your parents know other classmates' parents?... So that they sometimes meet up or phone	1	0.041	0.020	–	–
Parental contact		2	0.038	0.018	–	–
Parental contact		3	0.039	0.019	–	–
School violence	Which classmates seriously punched or kicked you, not for fun?	1	0.029	0.012	–	–
School violence		2	0.030	0.012	–	–
School violence		3	0.028	0.021	–	–
Mean of school violence		1–3	0.029	0.006	–	–
**Actor attributes**						
Girl (coCovar)	At least one parent immigrated	1	0.467	0.499	0	1
Immigrant (coCovar)		1	0.197	0.398	0	1
GPA (varCovar)	Grade point average in Maths, English, German	1	3.000	0.736	1	5
GPA (varCovar)		2	2.911	0.858	1	5
GPA (varCovar)		3	2.770	0.848	1	5
Parents: academic degree (coCovar)		1	0.443	0.497	0	1
No. students in class		24.80	3.326	20	30

Source: Own computation.

We first analyse the association between IC and violence in order to get a first impression on how closure corresponds with norm violations. In the second step, we investigate the co-evolution of children's friendship and parental contact networks. The co-evolution approach of stochastic actor-oriented models (SAOMs) (Steglich et al., 2010; Snijders et al., 2013) allows disentangling non-recursive effects: to some degree, children's friendships result from contact among parents, and to some degree contact among parents results from children's friendships. In both cases, we observe intergenerational closure, but since the mechanisms of how closure emerges are different, the effects of these two forms of closure might differ as well.

Our indicator of school violence results from a social network generator in the school survey asking ego whether alter has “seriously punched or kicked” him or her. We aggregated this information on the school class level by computing the *density* of the “punching and kicking” network, which is the outcome of interest in the first step (see Table 1 for the wording of the network generator).

The algorithm behind the SAOMs assumes actors who decide *as if* they were rational agents maximizing their utility by choosing network ties. It simulates micro-steps between the discrete-time measurements of networks. In each micro-step, only one actor makes his or her decision, which implies that the algorithm requires directed network-ties in its standard version (Steglich et al., 2010). Strictly speaking, contact among children's parents is not conceptualized as a directed network tie. As a measurement of parental contact, children reported the number of classmates whose parents are acquainted or have regular contact with their own parents. The wording of the item is “Do your parents know the parents of other classmates in your class? So well that they sometimes meet or talk on the phone?” Ego nominated the respective children into the network generator for this question. Due to measurement error, but also because children might perceive contact among parents in varying ways, reports on (non-)ties do not necessarily match between ego and alter. Technically, this mismatch is sufficient to create a directed network and to analyse it with the SAOM as a directed network.

More important than the technical applicability of this indicator is how students perceive contact among parents: only if they are *aware* of contact among their own and their peers' parents, they are able to report it, and contact they are aware of might have a stronger effect on their own behavior. Following from this, the network generator indicates each ego's awareness of their parents' contact to the peers' parents, which we regard as a crucial condition for ego's behavior. We control for network-structural effects (reciprocity, transitivity, in- and outdegree), “same (non-)immigrant status” (at least one parent immigrated), same gender and educational status in the analysis of network co-evolution. In the parents' network we control for “same gender,” “same (non-)immigrant status,” children's violent conflicts and “same (non-)academic degree,” while we also control for similarity in grade-point average (“gpa similarity”) in the friendship network.

We apply the Bayesian multilevel SAOM that combines the *k* = 10 networks and allows the estimation of random slopes, that is, varying strengths of effects across networks (Koskinen and Snijders, 2022). In addition to the intercept, we limit the random slopes to the variables of interest, namely the two mechanisms of closure, and (per default) the rate parameters. Table 1 shows the descriptive statistics and short names of the estimated SAOM effects (Ripley et al., 2020). The network densities of the outcomes predicted in the first step of our analysis—friendship and parental contact—vary over the three measurement occasions. The actor attributes “gender,” “immigrant” and “parents (non-)academic degree” are time-constant covariates (coCovar), whereas the grade point average (gpa) in German, Maths and English is time-varying (varCovar).

## 4. Results

**Figure 3:**
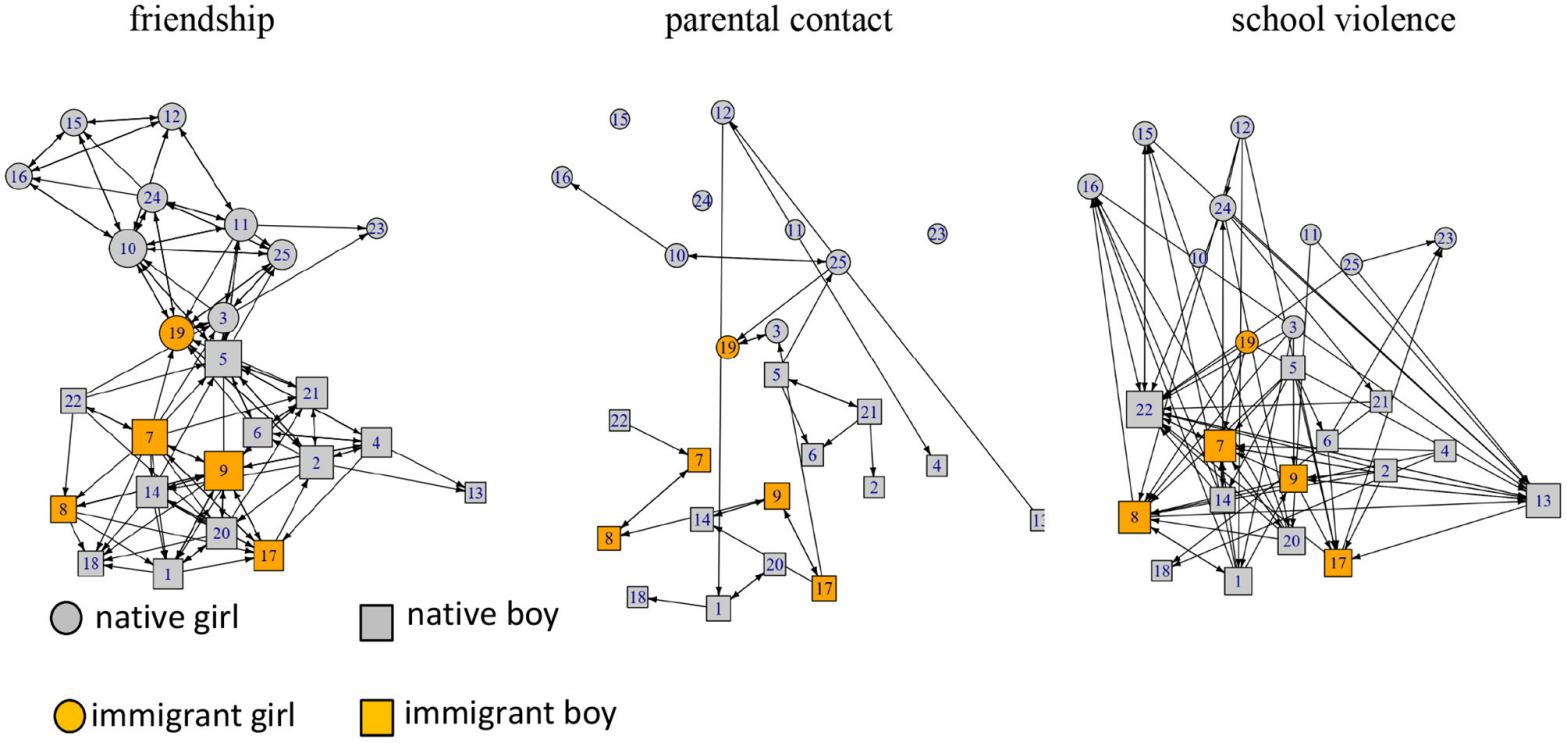
Example of network dimensions of “friendship,” “parental contact” and “school violence”.

Figure 3 shows three dimensions of a school class network: “friendship,” “parental contact” and “school violence.” For instance, nodes 7 and 8 as well as nodes 9 and 17 both agree that their parents are in contact and that they “sometimes meet or phone” (middle panel in Figure 3). In the school violence network (right panel in Figure 3) we find comparatively high in-degree for nodes 13 and 22, which means that both are often nominated as violent offenders. We assume that norm-enforcement results from contact among parents. At the level of classes as social systems, we expect that higher densities in parents' contact networks correspond with lower densities in school violence networks.

**Figure 4:**
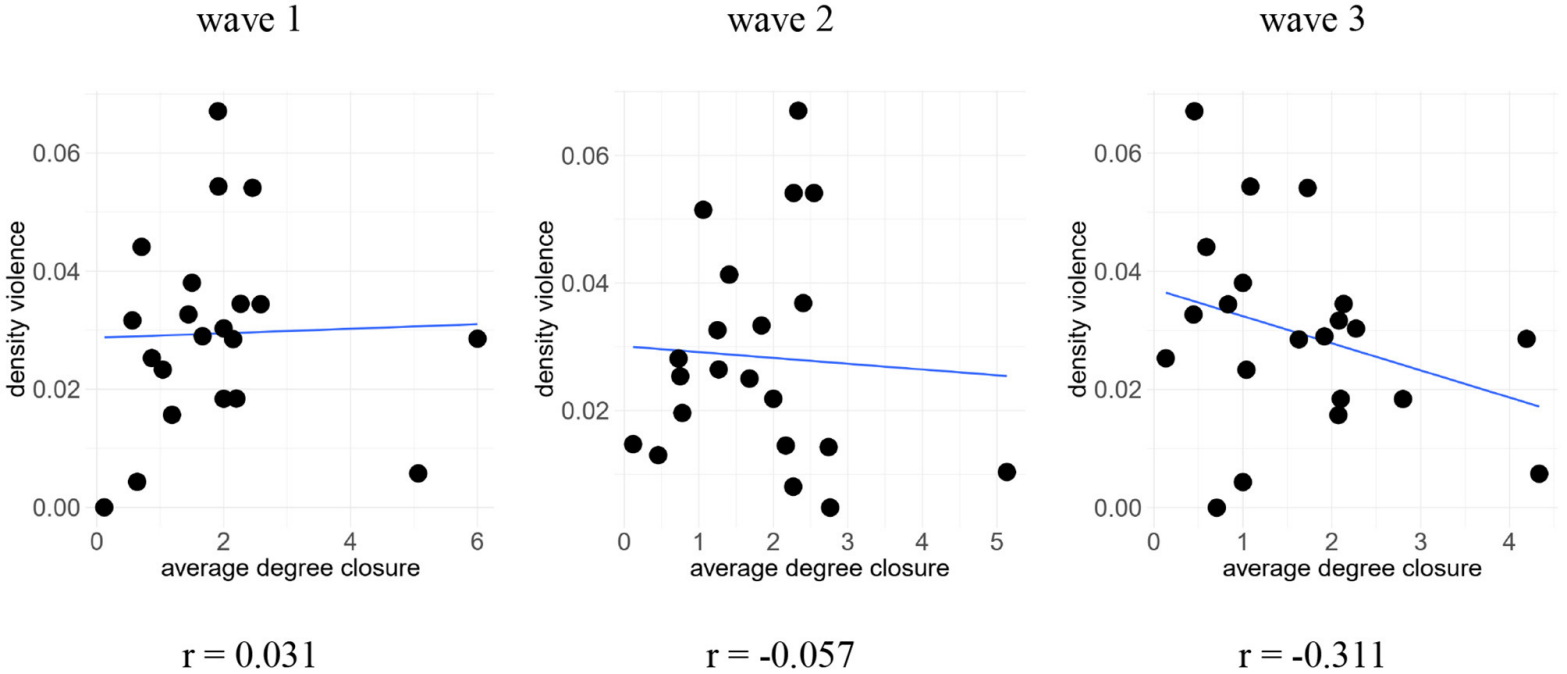
Correlation of global network characteristics: “Average indegree closure” and “school violence density,” *N* = 501 students; *k* = 21 classes. Source: Own computation.

In Figure 4 we computed the mean average degree for each of the *k* = 21 networks of “IC” and the densities of “school violence” over the three measurements from grade 5 to grade 7. Here, IC is a combination of two networks, namely parental contact and friendship among children. We multiplied both adjacency matrices, so that a cell in the resulting matrix contains 1 only if there is a 1 in the respective cell in *both* networks. According to Coleman's theory, this combination of both networks best captures the essence of IC because ties at both levels, children and parents, are necessary to generate IC.

Interestingly, the correlation changes over time: the positive association in wave 1 (*r* = 0.031) turns negative in wave 2 (*r* = −0.057) and wave 3 (*r* = −0.311). Given the small number of networks, the latter correlation is insignificant (*t* = −1.4309, *p* = 0.1687). Hence, based on a small number of networks, any interpretation of this correlation remains necessarily tentative, but it suggests that the association of IC and violent behavior needs some time to unfold from wave 1 to wave 3. This is what we would expect if there were indeed bottom-up closure: over time, children become befriended, parents become involved, but need some time to establish norms, which need some additional time to affect children's behavior.

Aside from that, violence among children can be a reason why parents get into contact if at least one party regards an intervention as necessary. Accordingly, our theoretical arguments suggest different mechanisms of intergenerational network closure, which should be disentangled in longitudinal analyses of networks.

Based on Bayesian multilevel SAOMs estimated for *k* = 10 classes and *N* = 248 students, the results of the co-evolution model with the two outcomes “friendship network” and “parental contact network” are shown in Table 2. In line with other social network studies, we find a strong and significant tendency toward reciprocity and transitivity (Windzio, 2018; Ripley et al., 2020) in children's friendship networks, which is the outcome in the first equation. Moreover, we find positive effects of “same (non-)immigrant status,” “same gender” and similarity in grade-point average (gpa). The latter is a control variable for educational inequality which is usually confounded with immigrant status. More important in our analysis is the effect of “parental contact,” which is positive, strong and highly significant on ties in students' friendship networks (0.667^***^). Here, “crprod” is the SIENA short name for the dyadic effect of having a tie in one network on having a tie in another network in a co-evolution model (Ripley et al., 2020, p. 146). The Bayesian multilevel approach allows the estimation of “random slopes,” that is, varying effect strength across networks of different classes. We introduced random variation for the intercept [outdegree (density)] and for the two effects of IC. According to the result, adolescents' friendship ties result to a considerable extent from contact among their parents, which is the “top-down” mechanism of how intergenerational closure emerges. In the second equation of the model, the outcome is the “parental contact network.”

**Table 2:** Co-evolution of adolescents friendships and contact among their parents, Bayesian multilevel SAOM *k* = 10 classes, *N* = 248 students.

**Effect**	**Par**.	**(Psd)**	**Betw. sd**.
**Effects on friendship network**			
Outdegree (density)	−3.504	(0.147)	0.154
Reciprocity	1.035	(0.048)	.
Transitivity (GWESP *I*-> *K*-> *J* (10))	1.952	(0.092)	.
Indegree-popularity	−0.011	(0.011)	.
Outdegree-activity	0.036	(0.003)	.
Gpa similarity	0.593	(0.099)	.
Same gender	0.514	(0.047)	.
Same (non-)immigrant status	0.145	(0.049)	.
Parental contact network (crprod)	0.667	(0.200)	0.167
**Effects on parental contact network**			
Outdegree (density)	−4.597	(0.483)	0.210
Reciprocity	1.887	(0.155)	.
Transitivity (GWESP *I*-> *K*-> *J* (5))	1.228	(0.246)	.
Indegree-popularity	−0.162	(0.074)	.
Outdegree-activity	0.136	(0.022)	.
Childrens' violent conflict (edge from network)	0.058	(0.267)	.
Same gender	−0.300	(0.147)	.
Same (non-)immigrant status	0.210	(0.140)	.
Same parents' (non-)academic degree	0.058	(0.109)	.
Friendship network (crprod)	2.865	(0.389)	0.178

Par, posterior mean; psd, posterior standard deviation; betw. Sd, posterior between-groups stand. deviation.

Source: Own computation.

One reason why parents get in contact might be children's behavioral conduct problems, so we include violence among children as an explanatory variable.^1^ However, we do not find a significantly positive effect of “violence among children” on ties in the parental contact network. “Same (non-)immigrant status” has a positive, but insignificant effect—at least, when the effect of children's friendship is controlled.

Furthermore, the positive effect of homophily with respect to parents' academic degrees is positive, although insignificant. We also find a strong, positive and highly significant effect of children's friendship ties on contact among their parents (2.865^***^), which yields insight into the second mechanism of how intergenerational closure emerges: friendships among adolescents and children in school classes have a strong and robust effect on contact among their parents. Although the estimated log odds are not directly comparable across equations due to the differences in baseline densities, intergenerational closure seems to be more strongly driven from “below” (2.865^***^ ≫ 0.667^***^). This is strong evidence of “bottom-up” IC.

Model convergence is a particularly important issue in SAOMs, also in the Bayesian multilevel specification. We ran the (time-consuming) model several times with different seeds for the random number and got almost identical results. In addition, Appendix 1 shows convergence plots for the overall model. Supplementary Figures A1–A6 should not indicate trends, but random variation around the estimate. There is a slight trend for the rate-parameters of friendship and parental contact from period 1 to period 2 (Supplementary Figure A5, Appendix 1). Although the estimation is not perfect, the other effects seem to have converged, so that the overall convergence for the *k* = 10 networks included in the multilevel SAOM analysis is at least acceptable.

The dataset includes *k* = 21 school-class networks (see Figure 4), but in a subset of 11 networks the density of ties among parents is so low that the SAOM convergence for the single network was too bad to include it in the multilevel SAOM. To estimate the effect we need to observe a sufficient amount of change in the parental contact network (Ripley et al., 2020), but the effect is not identifiable in a stable manner if the network density is too low. Of course, excluding a considerable number of observations for reasons related to the outcome of interest (ties in the parental network) is problematic with respect to random sampling and statistical inference. For instance, imagine a situation where density in children's friendship network is high but parents do not establish ties at all. This would be clear counter-evidence against the hypothesis of bottom-up closure, namely that ties among children would increase the probability of ties among parents. A different view on this issue is that an analysis of co-evolution needs sufficient information. We are interested in situations where sufficient information exists and given that, in whether children's and parents ties co-evolve. This procedure is similar to the “throwing away” of information in fixed effects panel regression (Halaby, 2004), that discards all information on differences between subjects in order to get the effect of changes within subjects. Likewise, SAOMs need a meaningful range of stability and dynamics in the networks over time to estimate the effects. If parental ties are out of this range, e.g., due to low density, we cannot use this information and it must “throw away” the data. In our view, this discussion is not yet settled, but we tend more to see the problematic aspects of excluding networks, even though there is not an alternative in studying IC based on complete networks. Here, we deal with sparse networks, namely contact among parents, which often do not converge smoothly.

To sum up, we find a negative correlation between IC and violent conflicts as a tentative result. The negative correlation needs some time to unfold, but remains still insignificant in wave 3. Despite of the small sample size, the dynamic modeling approach of the SAOM allows us to disentangle these two types of intergenerational closure. Coleman gave convincing arguments why community level social capital can reduce adolescents' delinquency and deviant behavior. However, neither Coleman himself nor researchers who conducted studies based on this concept distinguished between these two mechanisms. Our network study provides evidence of both mechanisms of IC—“top-down” and, potentially considerably stronger, “bottom-up.”

## 5. Conclusion

Coleman's concept of intergenerational closure is a powerful systematic and analytic theory of social capital and social integration among families. It is a multilevel theory of integration for at least two reasons: networks of children and parents mutually influence each other, and ties in subnetworks correlate with characteristics of the network as a social system. Studies apply Coleman's concept, but do not use complete social network data (Valasik and Barton, 2017; Geven and van de Werfhorst, 2020), even though Coleman explicitly developed IC as a network theory.

Although tentative, our results suggest that the emergence of ties among parents and, subsequently, the generation and calibration of norms takes some time, because the association between IC and the density of children's networks of violent interaction turns negative only in the third wave. Results are necessarily tentative since they are based on *k* = 21 observations (school classes) only. Evaluating the effects of IC on behavior implies switching the analysis from the actor- or dyadic-level to the level of the social system, in our case the school class. According to Coleman's theory, the more a social system tends to IC the easier it is for parents to generate and enforce norms. To become effective in a respective social system, however, norms must be a group-level rather than a 4-cycle or dyadic-level characteristic. Given that, the number of observations is often rather limited because there are not many longitudinal network studies that cover large numbers of networks. Moreover, as we have shown in Section 2, empirical results regarding the behavioral consequences of IC are rather ambivalent. Regarding our tentative result in combination with other studies (Windzio and Heiberger, 2022), we still suppose IC to be a potential factor that facilitates norm-generation and enforcement and reduces adolescents' violent behavior. Yet, this field needs further research based on larger data sets.

We distinguished between two mechanisms of intergenerational closure: according to the “top-down” mechanism, parents establish social network ties to other parents in their local community—due to their involvement in social or religious organizations. In contrast, the “bottom-up” mechanism suggests that contact among parents is a consequence of friendship-ties created by children and adolescents in schools and school classes. Our dynamic approach to social networks is based on stochastic actor-oriented models (SAOMs) and allows us to disentangle these mechanisms. Results provide evidence for the bottom-up as well as the top-down mechanism.

As already noted, the dataset we used for our analysis is small and we had to exclude data due to model non-convergence—which in turn is a consequence of low densities in the parental contact networks. The potential to generalize our results is thus limited. Moreover, albeit intergenerational closure has been measured as a directed network in our data, it is directed in a “cognitive” sense. Differences between ego's and alter's nomination result from differences of how both actors perceive the relationship among their parents. The directed measurement of individuals' self-reported perception of closure indicates how adolescents perceive social control. This perception determines the definition of the situation and guides the actor's behavior (Thomas and Znaniecki, 1996). Hence, we retained the common approach to analyse *directed* ties in the SAOM, because we do not see any conceptual advantage of applying SIENAs options to create undirected networks (Ripley et al., 2020, chp. 5.8). There are several ways of transforming directed ties to undirected ties, based on specific assumptions. Instead, we used the directed information, which is in line with the “cognitive” effect, namely the *perception* of social control. Future research should apply our measurement of closure in different social settings, collect data on more networks and analyse different outcomes, e.g., non-verbal violence, prevalence of substance use or the effect of ethnic group-specific closure on cultural boundaries between groups, indicated by the distribution of particular values and religious orientations.

To our knowledge, our study is the first that applies longitudinal social network data to distinguish between different mechanisms of intergenerational closure. This network structure is a crucial condition of a functional community, where “… the parent need not depend on the child itself for information about its behavior, in school and out. The parent has additional channels through the friends and acquaintances of the child, those children's parents, and back to the parent” (Coleman, 1987, p. 188). Future research on social integration and cohesion should systematically analyse these macro-micro interdependencies, and the effects of network structures on individual behavior.

## Data availability statement

The raw data supporting the conclusions of this article will be made available by the authors, without undue reservation.

## Ethics statement

This project involving human participants was reviewed and approved by the Bremen Senate for Education. Schools belong to the City State of Bremen and the City State decides on the approval after a severe review process concerning ethics, data privacy, and also political issues. Written informed consent to participate in this study was provided by the participants' legal guardians/next of kin.

## Author contributions

MW prepared the data analyses and manuscript preparation. PK was strongly involved in discussion of the IC approach, manuscript preparation, and refining the paper. Both authors contributed to the article and approved the submitted version.

## Footnotes

^1^The movie “Carnage,” based on a Yasmina Reza's intimate theater (“Le Dieu du carnage”), gives an impression of how violence among children can generate social interaction among parents.
