# Supplementary material for: The dynamics of intergenerational closure and family networks of social cohesion
Source: Front Sociol. 2023 Mar 2;8:933216. doi: 10.3389/fsoc.2023.933216 (PMC10018156; doi:10.3389/fsoc.2023.933216)
Supplement: Supplementary file 2 [file Data_Sheet_1.docx]

**Appendix 1**

| A1: Posterior effects, friendship equation, group constant effects | A2: Posterior effects, parents equation, group constant effects | |
| --- | --- | --- |
| 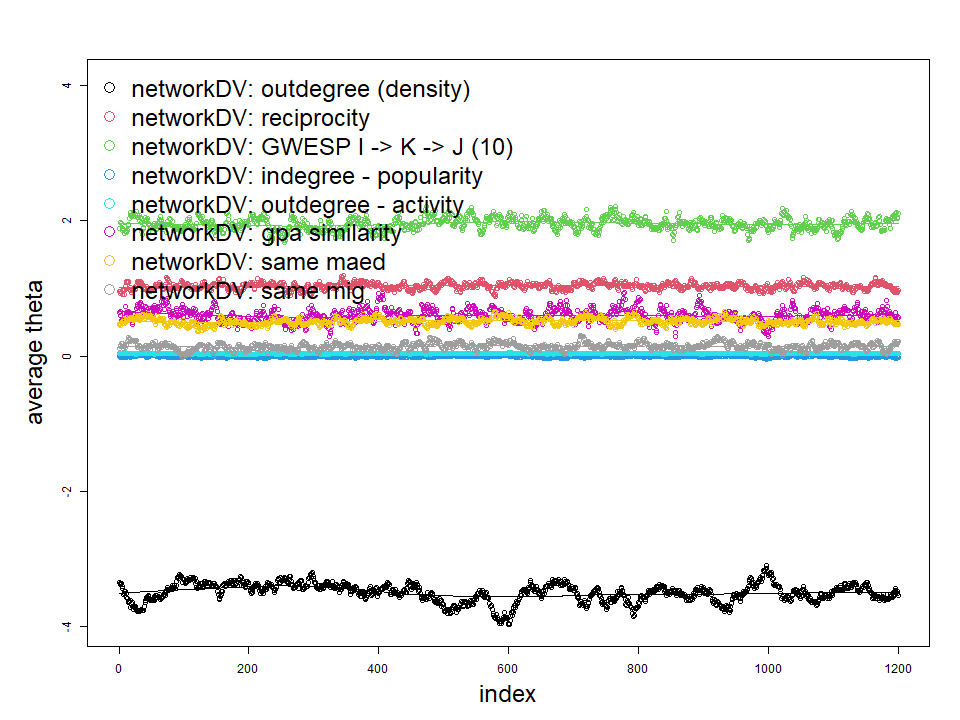 | 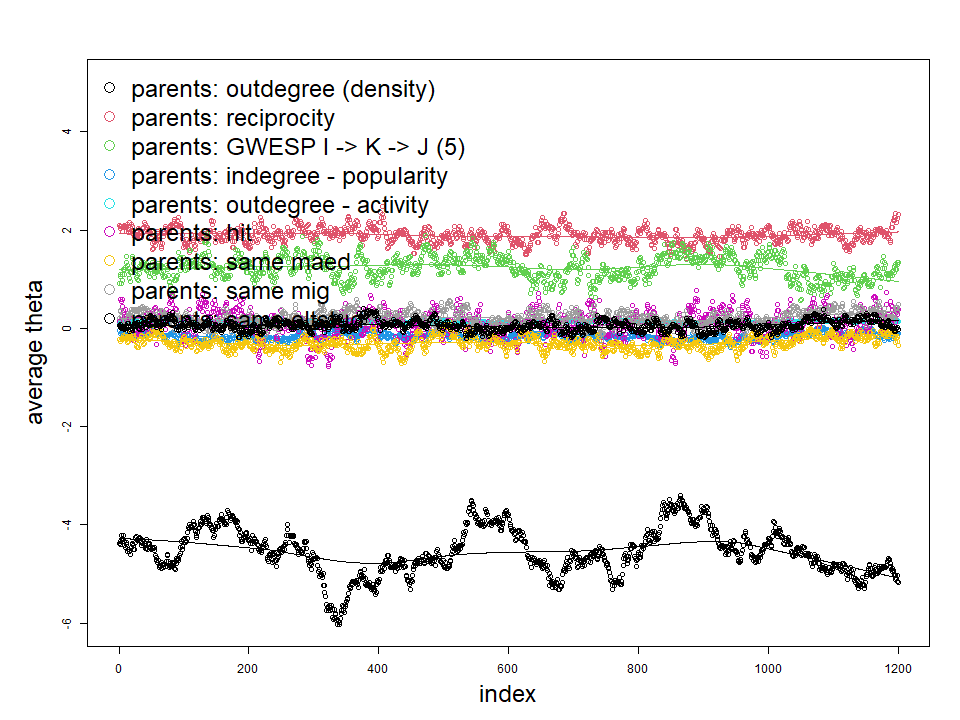 | |
|  |  | |
| A3: Posterior group varying effects | | A4: Posterior group varying effects, standard errors |
| 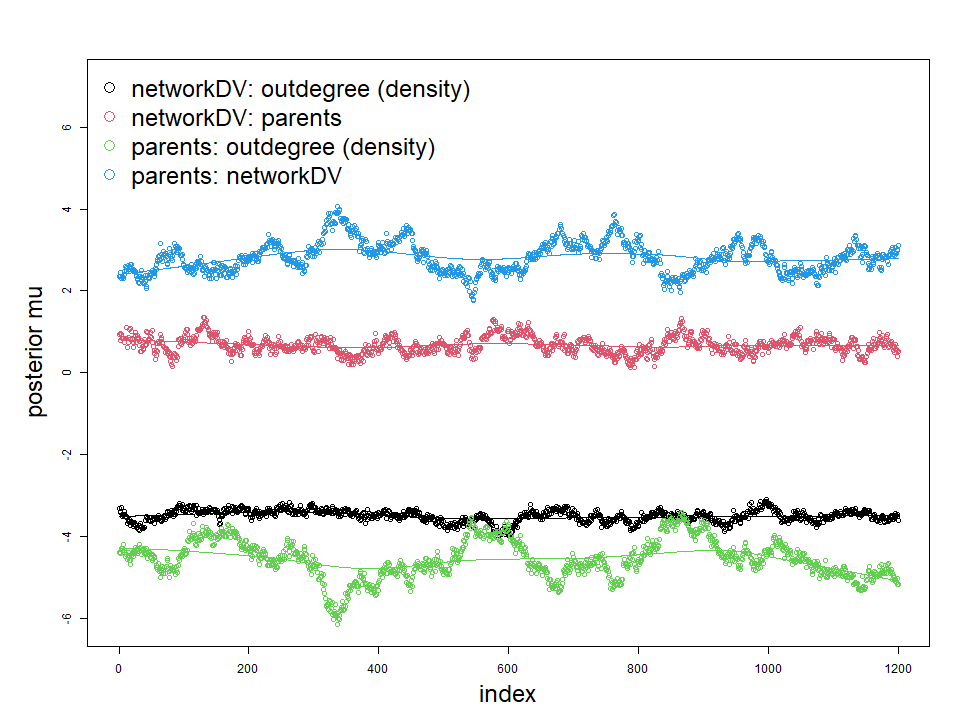 | | 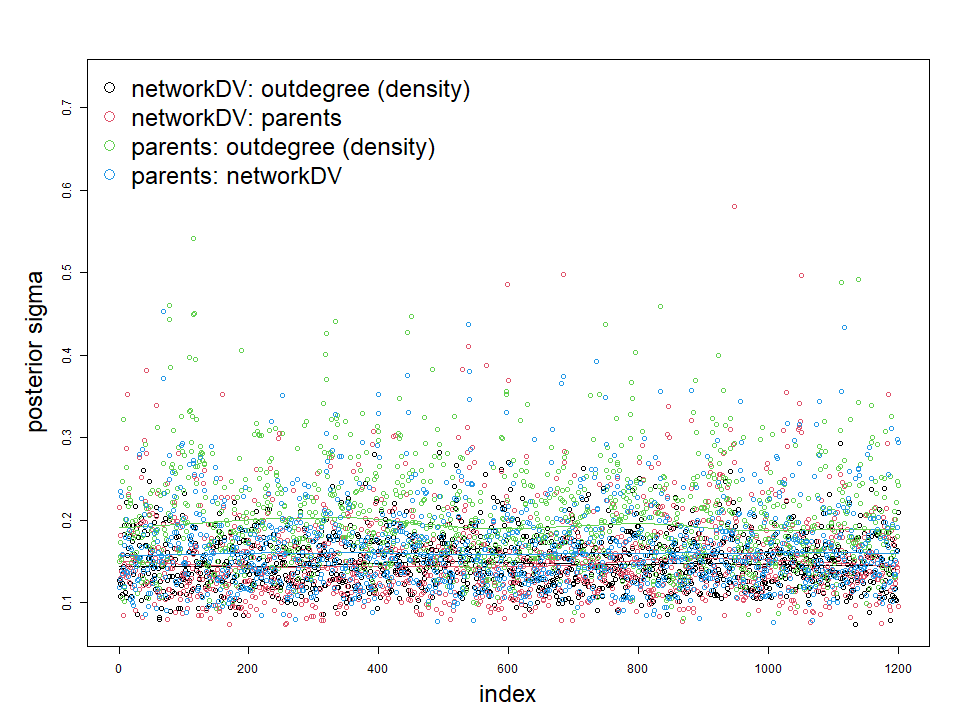 |
| A5: Posterior group varying rate effects | | A6: Posterior group varying rate standard errors |
| 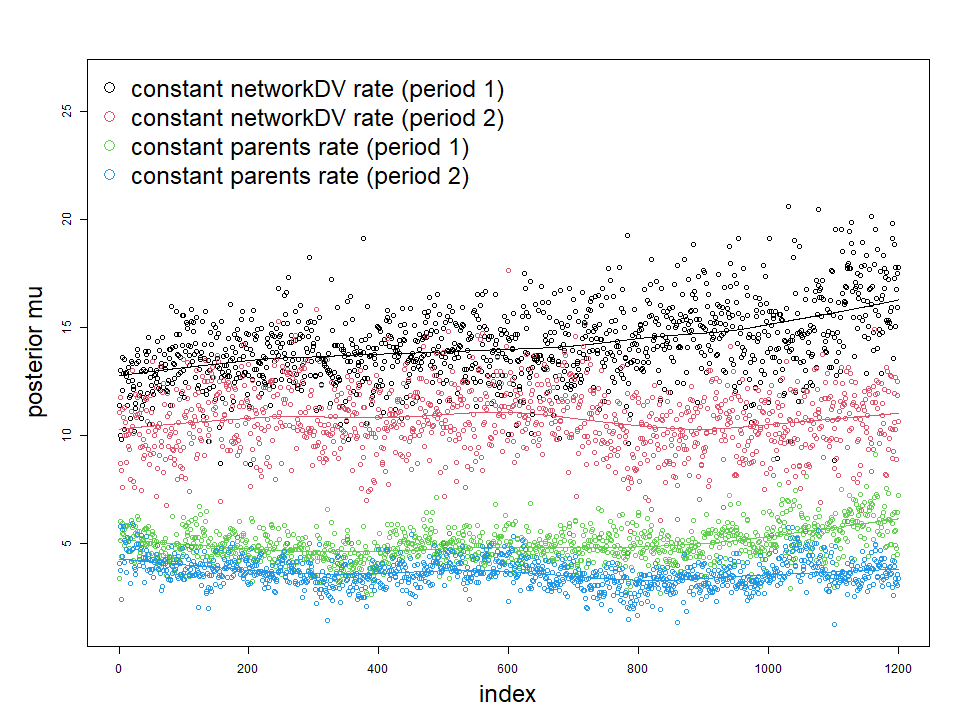 | | 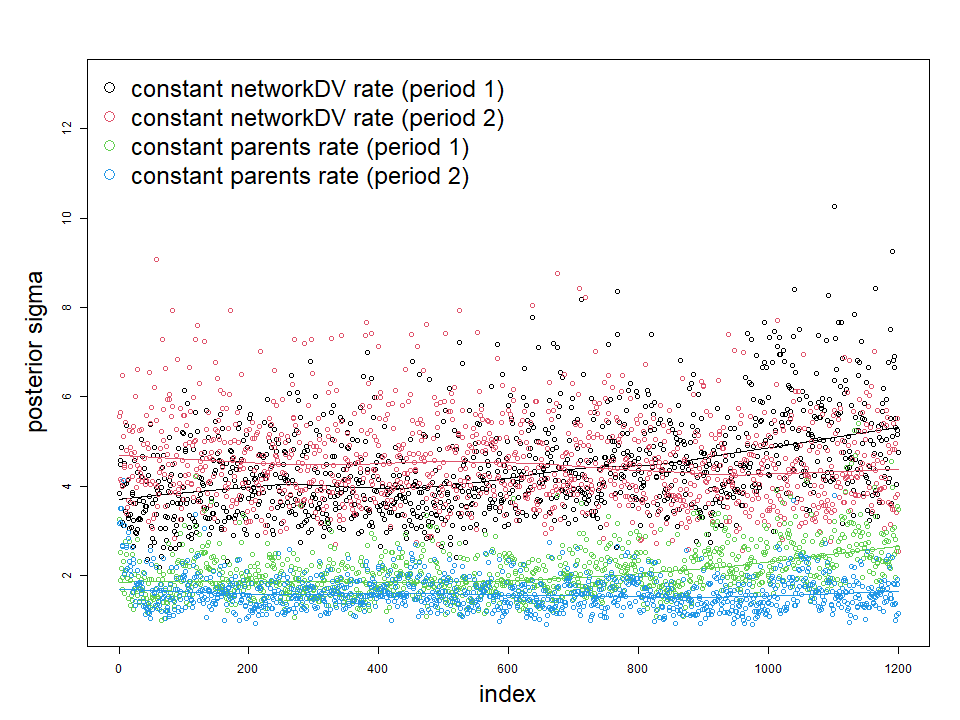 |

| A7: Density distribution of rate effects, friendship network | A8: Density distribution of rate effects, friendship network |
| --- | --- |
| 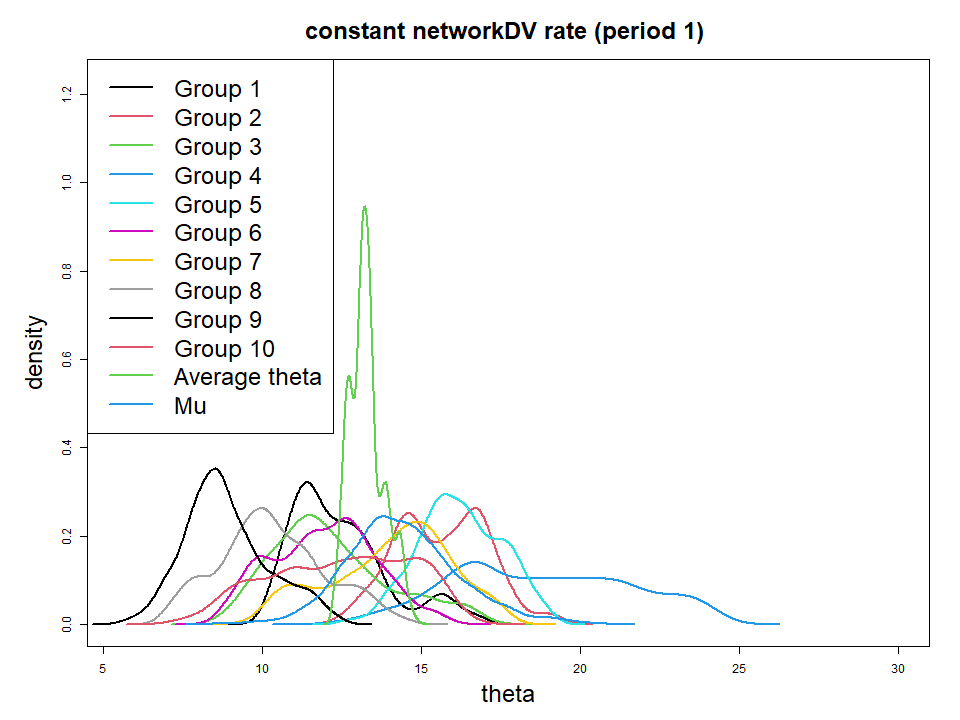 | 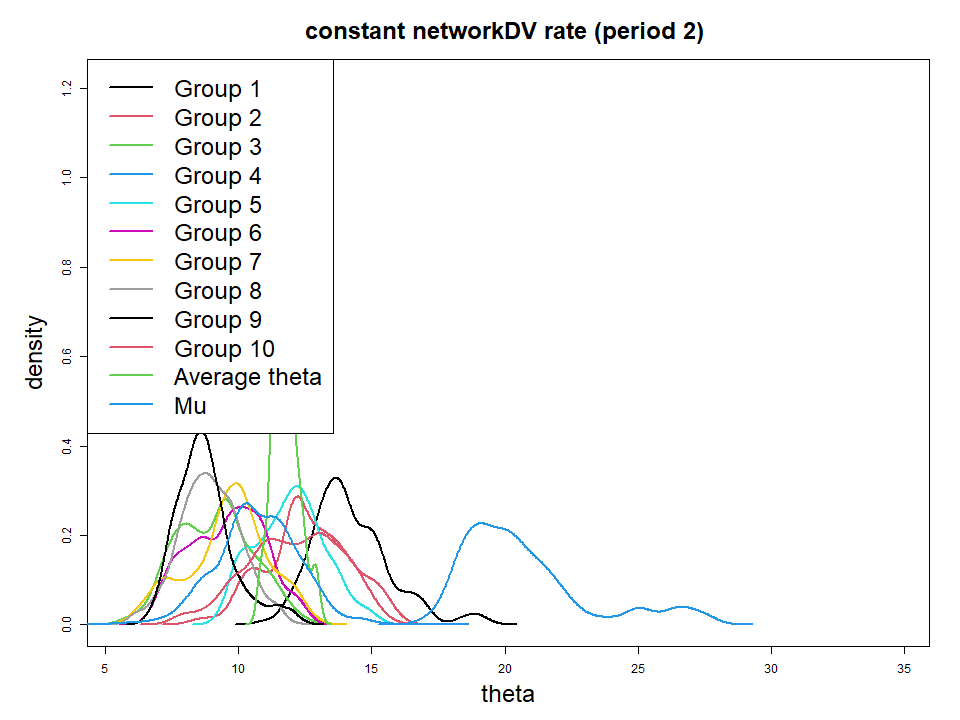 |

| A9: Density distribution of rate effects, parental contact network | A10: Density distribution of rate effects, parental contact network |
| --- | --- |
| 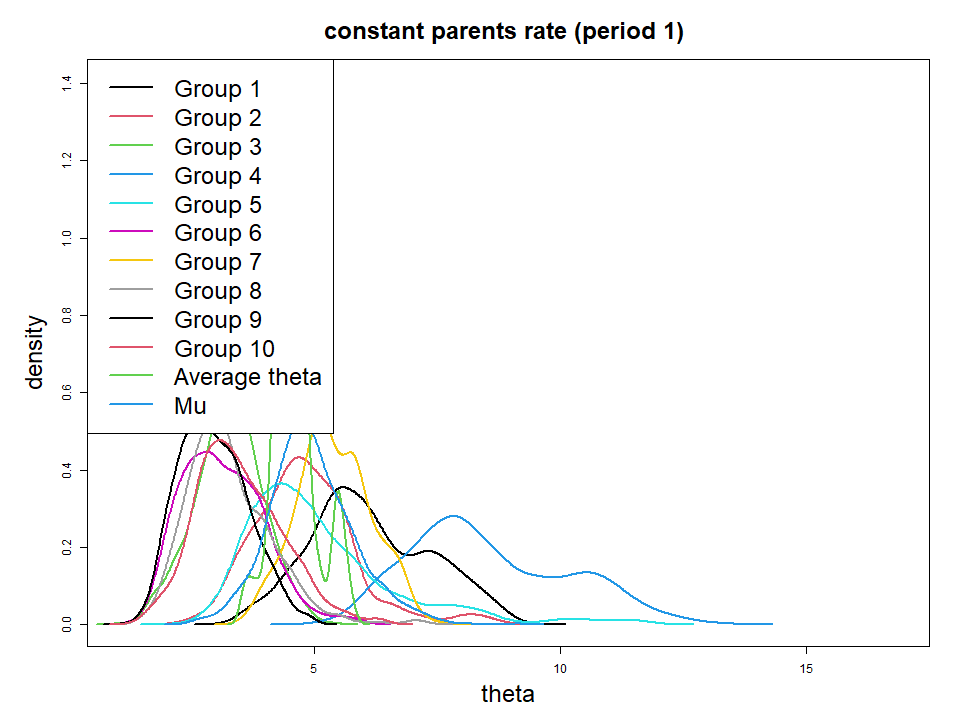 | 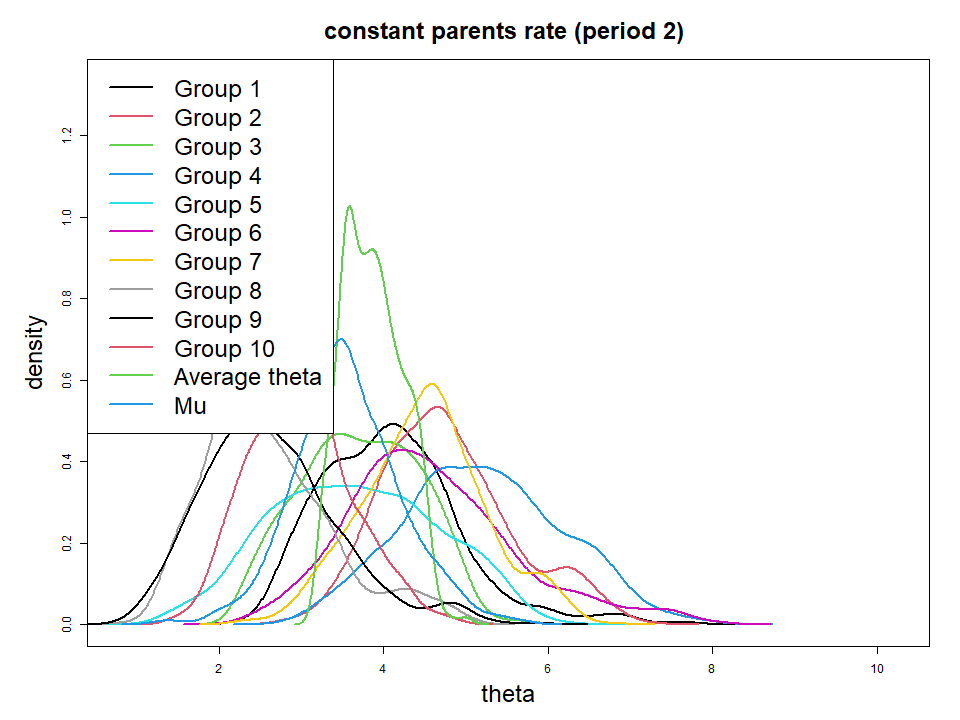 |
